# The Epidemiology of Dental Pathologies in Elderly Patients Admitted to a Tertiary Level Hospital in Oradea, NW Romania: A 5-Year Retrospective Study

**DOI:** 10.3390/healthcare11111522

**Published:** 2023-05-23

**Authors:** Michael Janto, Raluca Iurcov, Abel Emanuel Moca, Cristian Marius Daina, Rahela Tabita Moca, Lucia Georgeta Daina

**Affiliations:** 1 Doctoral School of Biomedical Sciences, University of Oradea, 1 Universității Street, 410087 Oradea, Romania; michael.janto@t-online.de (M.J.); rahelamoca@gmail.com (R.T.M.); 2 Department of Dentistry, Faculty of Medicine and Pharmacy, University of Oradea, 10 Piața 1 Decembrie Street, 410073 Oradea, Romania; 3 Department of Psycho-Neuroscience and Recovery, Faculty of Medicine and Pharmacy, University of Oradea, 10 Piața 1 Decembrie Street, 410073 Oradea, Romania; cristi_daina@yahoo.co.uk (C.M.D.); lucidaina@gmail.com (L.G.D.)

**Keywords:** elderly patients, dental pathologies, oral health, NW Romania

## Abstract

Oral diseases can affect the quality of life of all individuals, including elderly people. In elderly people, the associated general diseases can increase the risk of dental pathologies or can impact their treatment. The main aim of this study was to identify elderly patients with dental pathology out of the total number of patients admitted to the Department of Oral and Maxillofacial Surgery at a tertiary-level hospital in North-Western Romania. Another aim was to describe the characteristics of the patients included in this study, as well as to analyze data from patients with dental pathologies. In this retrospective study, the medical records of patients admitted to the Department of Oral and Maxillofacial Surgery at the Bihor County Emergency Hospital between 2016 and 2020 were analyzed, with a focus on patients aged 65 years or more. After applying the exclusion criteria, 721 patients were kept in the study, of which 316 (43.8%) had at least one dental pathology. Most elderly patients with dental pathologies were admitted in 2018 (*n* = 89). The most common associated systemic diseases were arterial hypertension (*n* = 268) and ischemic heart disease (*n* = 233), while the most common dental pathologies were pulpitis (*n* = 185), chronic apical periodontitis (*n* = 61) and abscesses (*n* = 35). Most patients were either healed or had an improved condition at the time of discharge. The great number of dental pathologies, as well as the diversity in dental pathologies, underline the necessity for better preventive programs aimed not only at children, adolescents or young people but at the elderly population as well.

## 1. Introduction

Oral health is important and influences the physical, psychological, emotional and social well-being of individuals, therefore having a multidimensional character. Unfortunately, oral health is frequently affected, and the prevalence of oral diseases is constantly increasing, rapidly turning into a real global public health problem [1]. Oral diseases are numerous and include dental caries, periodontal diseases and tooth loss [2], these being among the most common dental pathologies. Oral pathologies can be influenced by various socio-economic factors and can occur in all age groups, although some oral diseases are more predominant in a certain age category [3,4].

Dental caries predominate in children and adolescents [5], whereas periodontal diseases, edentulism and complications of dental caries (such as pulpitis, periapical periodontitis) predominate in the elderly population [6]. In addition, elderly people may have associated systemic diseases that may increase the risk of oral problems and may also influence their treatment [7]. The complexity of the vulnerability of the elderly, in terms of oral health, due to specific problems and particularities that correlate with general and loco-regional evolutionary issues, often requires a multidisciplinary approach to establish fair treatment in a hospital setting [8].

The provision of dental medical services in Romania is performed by dentists who work in private dental offices and clinics [9]. Based on data published at the national level, the number of dental examinations and treatments in Romania during 2011–2020 decreased by approximately 30%, which indicates a decrease in the addressability of patients to dental services providers. The National Health Insurance House reimburses expenses for dental services, but the budget allocated for oral health is low [10,11], not covering the needs of patients.

The hospital service that provides dental care in Romania is the oral and maxillofacial surgery department, where doctors with this specialization provide medical services for treating diseases in the maxillofacial area [12].

The prerequisite for this study was the existence of limited epidemiological and clinical data on elderly patients requiring public dental medical services. It is of major importance to understand dental pathology in elderly patients in order to be able to provide excellent medical services in terms of diagnosis and treatment. In addition, diagnosing oral pathology in the elderly acquires special importance, thus making it necessary to know the general health status of an elderly patient using a correct and complete assessment of the basic condition and associated comorbidities. The quality of life of elderly patients with dental pathologies must be approached transdisciplinary and interdisciplinary [13].

The public healthcare system in Romania makes it difficult for patients with dental pathologies to access public hospital services, given the fact that non-emergency cases must have a referral from a general practitioner doctor. Considering the complexity and variety of dental pathology encountered in the elderly patient [14], this research aimed to identify the number of elderly patients with dental pathology out of the total number of patients admitted to the Department of Oral and Maxillofacial Surgery at a tertiary-level hospital in Oradea, a city located in North-Western Romania. Another aim was to describe the characteristics of the patients included in this study, as well as to analyze data from patients with dental pathologies.

## 2. Materials and Methods

### 2.1. Ethical Approval

This study was approved by the Ethical Council (IRB No. from 11 February 2021) and the Ethical Committee (IRB No.4364 from 12 February 2021) of the Bihor County Emergency Hospital. In conducting this study, all policies and recommendations stated in the 2008 Declaration of Helsinki and its later amendments were respected. All patients signed an informed consent, agreeing to future anonymous use of the information contained in their medical records.

### 2.2. Participants and Data Collection

This study was designed as a retrospective study, and it involved the analysis of medical records belonging to patients who were hospitalized in the Department of Oral and Maxillofacial Surgery at the Bihor County Emergency Hospital. The hospital is located in Oradea, the biggest city in Bihor County. This is a tertiary-level hospital with various departments. The Department of Oral and Maxillofacial Surgery treats patients with cases of medium complexity. All medical records belonging to patients hospitalized between 2016 and 2020 were analyzed. The patients were admitted for pain, swelling, poor general health status due to oral and maxillofacial infection, facial trauma, treatment of benign or malignant tumors and dentoalveolar and maxillofacial surgery.

The following inclusion criteria were taken into consideration: hospitalized patients with dental pathology; patients aged 65 years or more and patients hospitalized in the Department of Oral and Maxillofacial Surgery. The following exclusion criteria were applied: patients younger than 65 years and patients for whom important information was missing from the medical record (e.g., age, gender, diagnosis).

Several variables were investigated and used to obtain important results. These variables were: age (65–69 years, 70–79 years, over 80 years); gender (male, female); living environment (urban, rural); associated systemic diseases (more comorbidities were identified); existing intra-oral status (various dental diseases or dental treatments that were not acute and did not represent the main reason for hospitalization) and dental pathologies (various dental diseases that represented the main reason for hospitalization). The therapeutic procedures and case evolution up until the moment of discharge were also identified and noted. Regarding patients’ evolution, the cases that did not present any positive or negative changes during the period in which the patients were hospitalized were considered stationary. The patients that had reduced clinical symptomatology (such as the considerable reduction in pain and swelling reduction) and whose laboratory results showed improvements that allowed their safe discharge were considered improved. Patients who no longer had any clinical symptoms at discharge, and for whom the laboratory results showed the absence of any inflammation, were considered healed. 

Oral hygiene was determined by the resident doctor who first examined the patients, and it was based on the simplified oral hygiene index (OHI-S), which describes three possibilities, these being good oral hygiene, fair oral hygiene and poor oral hygiene [15]. Attrition, abrasion and erosions were considered as being different types of tooth wear [16].

The original medical files were completed and signed both by a resident doctor in the specialty of oral and maxillofacial surgery and by the specialist doctor of oral and maxillofacial surgery. All medical records were double-checked by two authors to avoid any potential bias.

### 2.3. Sample Size Estimation

According to the data from the last census carried out in Romania in 2021, the population over 65 years is represented by 3,726,460 people. This represents approximately 20% of the total population of the country [17]. Based on these values, it was calculated that in order to obtain a confidence level of 95%, the sample size should be at least 246 patients so that the real value is within 5% of the measured values. However, we included in the study all the patients who remained after applying the inclusion and exclusion criteria.

### 2.4. Statistical Analysis

SPSS Statistics for Windows (IBM, Chicago, IL, USA) was used to analyze the data collected in this study. Data that showed a normal distribution were expressed as a number (*n*) or as a percentage (%). For the comparative analysis and statistical validation of the results, a “*t*-test” was used. *p* with a value <0.05 was considered statistically significant.

## 3. Results

### 3.1. Distribution of Elderly Patients

**Figure 1:**
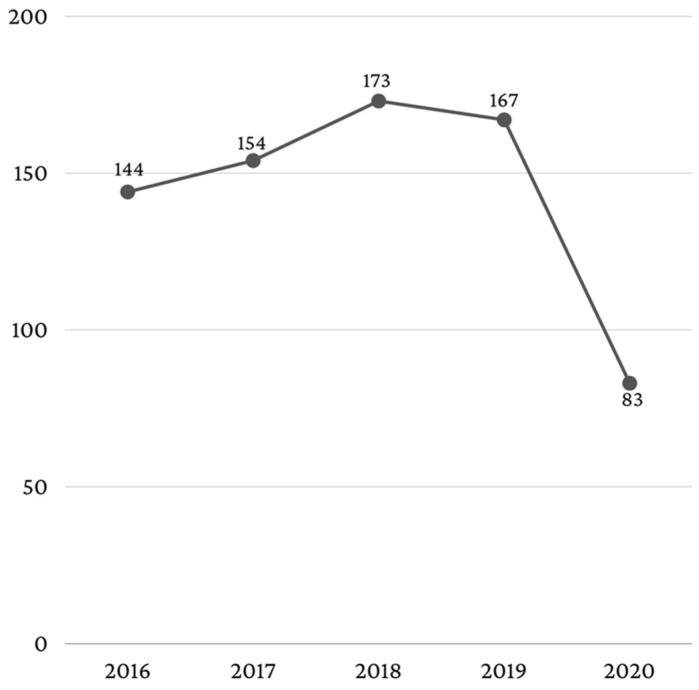
Number of elderly patients per year.

During the analyzed period, a total of 3391 patients were hospitalized, but after applying the exclusion criteria, a final number of 721 patients aged ≥65 years remained in this study, representing 21.3% of the total number of patients hospitalized during the 5 years investigated. During the 5 years, the share of patients ≥65 years old had a sinusoidal evolution, with a minimum in 2020 (*n* = 83, 11.5%) and a maximum in 2018 (*n* = 173, 24%) (Figure 1).

Out of the total number of elderly patients, 359 (49.8%) were male patients, and 362 (50.2%) were female patients. Therefore, female patients predominated in the studied group, but the differences between genders were not statistically significant (*p* = 0.973). Regarding the living environment, 341 (47.3%) patients were identified as living in a rural environment and 380 in an urban environment (52.7%). The *t*-test showed that the number of patients who lived in an urban environment was significantly higher than the number of patients who lived in a rural environment (*p* = 0.002) (Table 1).

### 3.2. Dental Pathologies in Elderly Patients

**Figure 2:**
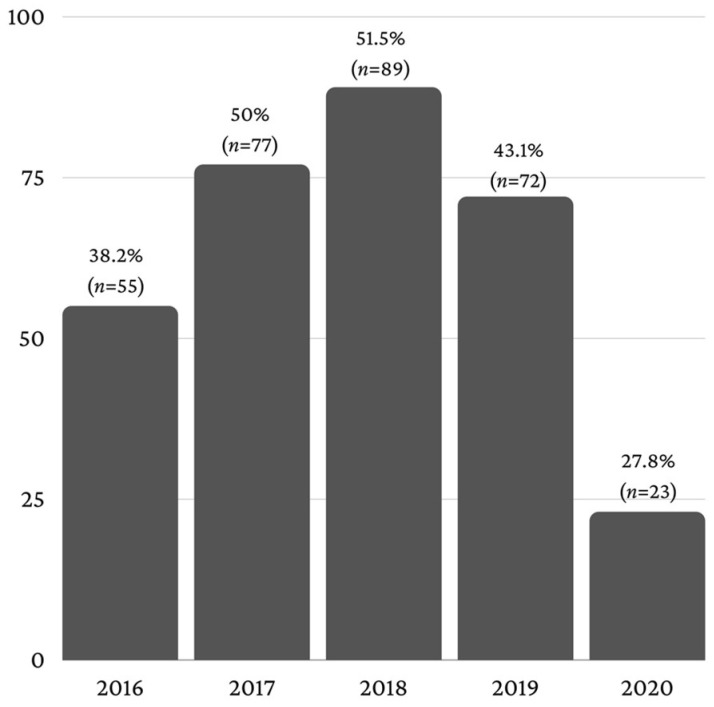
Elderly patients with dental pathologies.

Of the 721 patients aged ≥65 years, 316 (43.8%) had a diagnosed dental pathology, of whom 170 were male patients (53.8%) and 146 were female patients (46.2%). The number of patients diagnosed with dental pathologies in the period 2016–2020 had an upward trend until 2018 (51.5%), after which a decrease was recorded, reaching a minimum in 2020 (27.8%) (Figure 2).

The average age of elderly patients showed small variations in the analyzed years, with values between 72.37 ± 5.02 years in 2018 and 73.75 ± 4.72 years in 2017, the average age being 73.07 ± 5.04 years. The average age of patients ≥65 years with dental pathologies was 73.07 years, insignificantly lower in male patients than in female patients (*p* = 0.267). Regarding the distribution of patients in the three age groups, it was observed that patients from the 70–79-year-old group predominated in the studied sample (49.7%, *n* = 157) (Table 2).

**Figure 3:**
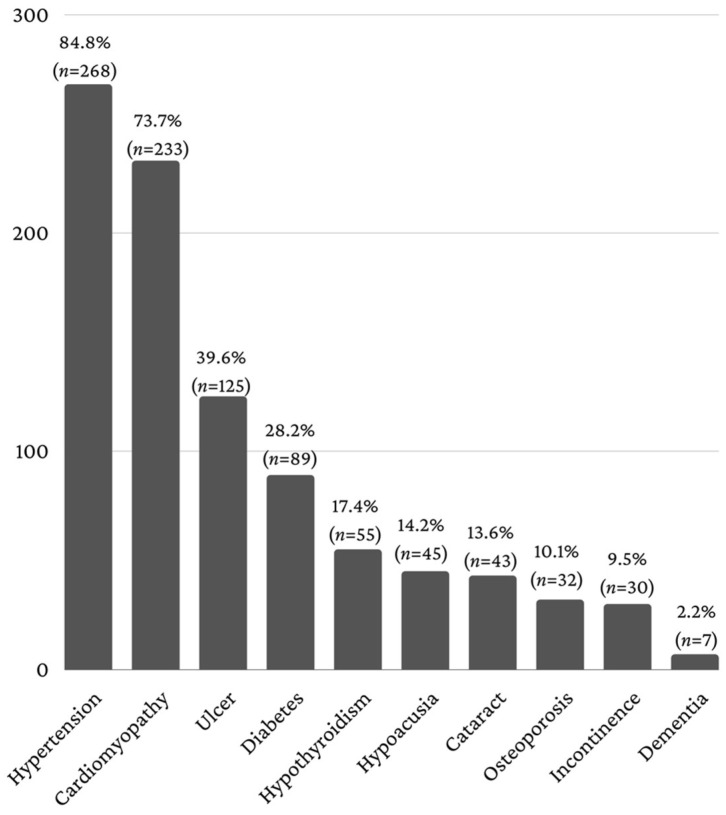
Associated systemic diseases.

Various associated systemic diseases were identified, the most frequent being arterial hypertension (84.8%, *n* = 268) and ischemic heart disease (73.7%, *n* = 233) (Figure 3).

**Figure 4:**
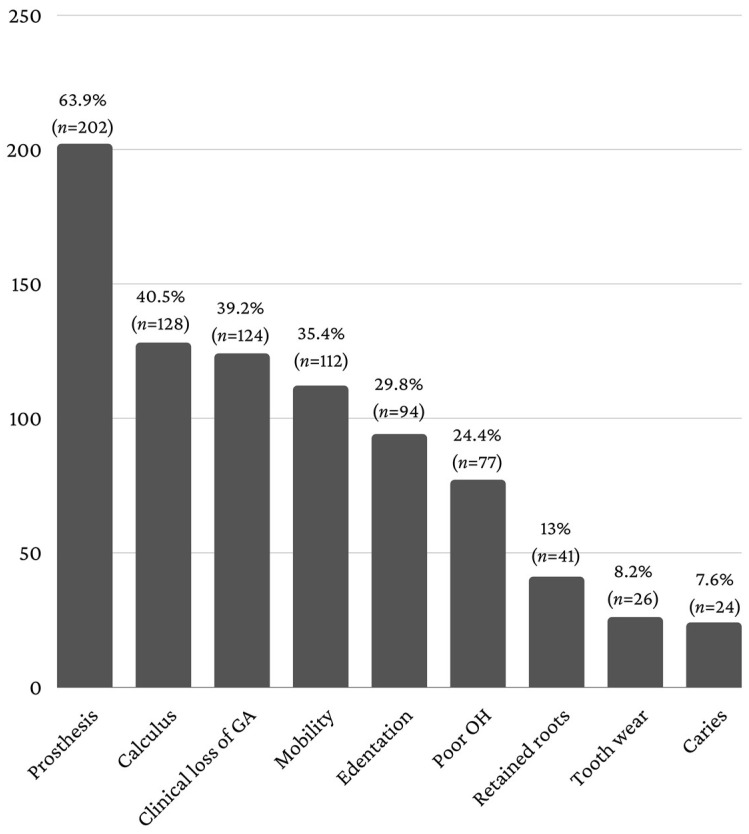
Intra-oral status.

The clinical examination revealed that more than 63% of the patients presented dental prosthetic treatment (63.9%, *n* = 202). Furthermore, dental calculus was recorded in 40.5% of the patients (*n* = 128), dental mobility in 35.4% (*n* = 112), clinical loss of gingival attachment in 39.2% (*n* = 124) and edentations in 29.8% (*n* = 94) (Figure 4).

The main diagnosis for elderly patients with dental pathologies is shown in Table 3. The most common pathologies were represented by pulpitis (58.5%, *n* = 185), chronic apical periodontitis (19.3%, *n* = 61) and abscesses (11.1%, *n* = 35). These pathologies were more frequently diagnosed in male patients, but not in a statistically significant matter (*p* > 0.05). Tooth fracture and tooth dislocation were recorded in three patients who suffered trauma from falling.

Depending on the clinical examination, paraclinical examinations and the main diagnosis, elderly patients benefited from specific procedures, as shown in Table 4. The most frequent procedures performed were alveolar bone surgery for fewer than eight teeth (68%, *n* = 215) and incision and drainage of an oral abscess or oral cyst (10.8%, *n* = 34).

The discharge status of elderly patients with dental pathology varied from one year to another. Most healed patients were discharged in 2016, 2019 and 2020, while in 2017 and 2018, most patients were discharged with an improved health status (Table 5). There were no registered deaths during the analyzed period.

## 4. Discussion

The focus on oral health has grown in recent years due to the awareness of the growing burden of dental diseases among the population. The WHO estimates that 90% [18,19,20,21,22] of the world’s population suffers from at least one oral disease during their lifetime. The elderly population is one of the age groups strongly affected by oral pathologies [14], and the investigation of this category of patients is important. It was considered that advancing age, gender of the patients, living environment and associated systemic diseases can impact the number and characteristics of dental pathologies and the therapeutic procedures applied, as well as the evolution of the cases. Thus, these variables were investigated. In addition, because this population group is rarely investigated, the number of patients with oral pathologies was also analyzed. 

The large differences between different countries regarding public coverage and accessibility of oral health services were the prerequisites for adopting the Resolution on Oral Health by the WHO in 2021 [23]. The aim of this resolution was to implement a universal oral health coverage plan in the member states. It is estimated that only one-third of dental care costs are covered by public sources [23], which makes dental care unaffordable for a large sector of the population, so most dental diseases remain unknown and untreated.

In Romania, dental offices can be private or public. Public dental offices can be school dental offices, dental offices that are linked to a dentistry school, dental offices that are linked to various institutions and dental emergency offices that belong to the Emergency Reception Unit at various hospitals [24]. Of the total number of dental offices, only 5% are public and cost-free, which is insignificant to the whole population, especially in rural areas, where their number is very small [25].

Dental pathologies are largely preventable with relatively low costs if timely and properly managed. As for the elderly population, dental procedures require significant costs both for the public health system and the individual. Even though the provision of dental services is available to patients through dental offices and clinics, the poor public financial coverage of these services causes patients to seek emergency treatment in a hospital unit. In Romania, elderly patients with dental pathologies access hospital care when they experience a worsening in their oral health due to either acute symptoms or chewing problems. In most cases, these patients have, in addition to the basic condition, a series of complications and comorbidities, which require a complex assessment of the general health status using a multidisciplinary approach [26,27,28].

The analysis of the epidemiological and clinical particularities in hospitalized elderly patients was performed in a tertiary-level hospital. The medical services provided in the oral and maxillofacial surgery department are of medium complexity. For a more complex analysis, the study period was 5 years (2016–2020). The number of hospitalized patients did not show large variations from one year to another, except for 2020, when due to the COVID-19 pandemic, hospitalizations were limited for many months only to cases of emergency [29,30,31,32,33].

The percentage of elderly patients out of the total number of hospitalized patients was 21.3%, of which elderly patients with dental pathology accounted for 43.8%. Considering elderly patients with dental pathologies to the total number of hospitalized patients, the result was 11.8%, which is close to the data published by the Romanian National Institute of Statistics in 2021, where people who were 75 years and older, visited the dentist in a proportion of 8.8% [34]. Other published studies also highlight the low usage of dental care by the elderly [35,36,37]. 

In Romania, the elderly population that benefits from dental treatment is represented by mainly male patients [27,28,29,30,31,34], and these results are similar to the present study, where most hospitalized elderly patients who had a dental pathology were male patients. The much easier accessibility to health care services for patients from urban areas was also reflected in the obtained results, where the number of patients with dental pathologies living in an urban environment (60.1%) was statistically significantly higher than the number of patients living in a rural environment (39.9%). Ogunbodede et al. (2015) reported that the accessibility to dental services for patients living in an urban environment in various African and Middle Eastern countries was higher, which is a result similar to our study, as well [38]. Results similar to our study were also obtained from populations in Bosnia and Herzegovina [39] and China [40]. This is probably due to the fact that people living in rural areas tend to dismiss the use of healthcare services and dental healthcare services [38]. In addition, 53.8% of the Romanian population lives in an urban environment [41].

Given the fact that an important feature of the elderly patient is comorbidity, the coexisting comorbidities in elderly patients with dental pathology were also analyzed. Comorbidity is one of the most important public health issues due to the complexity of therapeutic management and the important financial implications. Once certain conditions have been diagnosed, a newly diagnosed pathology in the same patient raises important problems in therapeutic conduct regarding drug interactions and side effects [42,43,44]. Most elderly patients with comorbidities have an altered state of health [44,45,46]. Cardiovascular diseases predominated in elderly hospitalized patients (hypertension—84.81%, ischemic heart disease—73.73%), followed by gastric/duodenal ulcer (39.56%) and diabetes mellitus (28.16%), and these data agree with published studies [47,48,49,50]. Impairment of functional capacity was present in elderly patients hospitalized for diseases such as osteoporosis, senile dementia, hearing loss, cataracts and diabetic retinopathy and sphincter incontinence. An intra-oral examination is an important component for establishing an oral diagnosis, giving information regarding the health status of soft and hard tissues [48,49,50,51,52,53]. Most of the hospitalized elderly patients presented prosthetic works (62.9%), dental calculus (40.5%) and clinical loss of gingival attachment (39.2%). Hospital morbidity due to dental diseases is consistent with the reports on diagnostic codes made by similar hospitals [47,48,49,50,51,52], with the most common diagnoses reported being pulpitis (58.5%), chronic apical periodontitis (19.3%) and abscess (11.08%).

The data obtained in our study do not fully allow a comparative analysis with data from the literature, where most studies identify oral health problems in institutionalized elderly patients. A similar investigation was carried out in 2018 in Nigeria on a sample of 106 elderly patients, the male/female ratio being 1:1.2 and the average age 68.7 ± 6.5 years, with a predominance of periodontal diseases and dental caries [54]. Another study conducted in two hospitals in Australia highlighted poor oral hygiene of hospitalized patients with no significant improvement after 7 days of hospitalization [55]. The need for future studies to stratify oral care intervention in elderly patients according to oral health conditions is emphasized [56].

Almost a quarter of the patients that were hospitalized during the investigated period were aged ≥65 years, of which almost half had a diagnosed dental pathology that was the main reason for presenting to the hospital. The vast majority of patients had at least one associated systemic disease. The dental pathologies were multiple, but following the application of different therapeutic protocols, most patients had an improved health status or were healed at the time of discharge. The access to dental treatment for the elderly population is reduced in Romania due to the fact that 90% of dental offices are private, and the state covers only a small amount of the costs compared to the needs of the population [24]. It is the authors’ belief that this study contains valuable information regarding the oral status of elderly patients and highlights the necessity for better healthcare plans for the elderly population. Preventive programs, aimed at reducing any oral pathology in the elderly population should be developed. This study also gives important information regarding the epidemiology of oral pathologies in patients aged 65 years or more.

However, this study has some limitations. First, the research was conducted in a tertiary care hospital from NW Romania, and the information could vary in different medical centers in Romania. It is possible that in other regions of Romania, the elderly population presents other characteristics, and the results presented in this study could be different from other areas of the country. For this reason, a multicentric approach that presents general national results would be welcome. Furthermore, the hospital where this study was conducted covers several specializations, not just oral and maxillofacial surgery. There are certain hospitals in Romania that are dedicated only to oral and maxillofacial surgery, so the number of patients and the characteristics of the identified pathologies could be different. However, in Bihor County, this is the only hospital that has an oral and maxillofacial surgery department. The nearest maxillofacial surgery hospitals are at a distance of over 100 km from the city of Oradea and are located in other counties. The number of hospitalized patients is not very high, but it is worth mentioning that the authors only considered elderly people.

## 5. Conclusions

Most patients included in this study were female patients who presented a variety of associated comorbidities, but the most common associated systemic diseases were hypertension and cardiomyopathy. Pulpitis, apical periodontitis, abscesses or cysts were among the diagnosed dental pathologies, and after treatment, most patients had an improved or healed condition at discharge. The elderly population is affected by dental pathologies, and the associated systemic diseases can make the treatment and evolution of these diseases negative. It is necessary to investigate this population at a national level in order to gain an overview of this age group and to be able to lay the foundations for national preventive programs. Providing periodic free consultations would be an important step to prevent the worsening of dental pathologies in the elderly.

## Figures and Tables

**Table 1:** Distribution of patients according to gender and living environment.

	Male		Female		*p* *
	No.	%	No.	%	
2016	68	47.2%	76	52.8%	0.973
2017	82	53.5%	72	46.5%	
2018	85	49.1%	88	50.9%	
2019	79	47.3%	88	52.7%	
2020	45	54.2%	38	45.8%	
	**Urban**		**Rural**		
	**No.**	**%**	**No.**	**%**	
2016	73	50.7%	71	49.3%	0.002
2017	75	48.7%	79	51.3%	
2018	97	56.1%	76	43.9%	
2019	95	56.9%	72	43.1%	
2020	40	48.2%	43	51.8%	

No., number; %, percentage; * *t*-test.

**Table 2:** Distribution of patients with dental pathologies according to age and gender.

Age	Total		Male		Female	
	No.	%	No.	%	No.	%
65–69 years	107	33.86	62	36.47	45	30.82
70–79 years	157	49.68	82	48.24	75	51.37
≥80 years	52	16.46	26	15.29	26	17.81
Mean age	73.07 ± 5.04		72.78 ± 5.04		73.41 ± 4.99	

No., number; %, percentage.

**Table 3:** Diagnosed oral pathologies.

Diagnosis	Total		Male		Female		*p* *
	No.	%	No.	%	No.	%	
Abscess	35	11.1%	19	11.2%	16	11%	>0.05
Gum diseases	7	2.2%	2	1.2%	5	3.4%	
Pulpitis	185	58.5%	101	59.4%	84	57.5%	
Dental cysts	6	1.9%	4	2.4%	2	1.4%	
Retained teeth	10	3.2%	2	1.2%	8	5.5%	
Fracture	2	0.6%	1	0.6%	1	0.7%	
Dislocation	1	0.3%	1	0.6%	0	0%	
Acute apical periodontitis	3	1%	3	1.8%	0	0%	
Chronic apical periodontitis	61	19.3%	35	20.6%	26	17.8%	
Retained root	4	1.3%	2	1.2%	2	1.4%	
Malign tumor	2	0.6%	0	0%	2	1.4%	

No., number; %, percentage; * *t*-test.

**Table 4:** Therapeutic procedures.

Procedures	No.	%
Biopsy	2	0.7%
Cyst excision	3	1%
Excision of an oral cavity lesion	4	1.2%
Alveolar bone surgery for ≤eight teeth	215	68%
Tooth extraction	2	0.7%
Surgical removal of two or more erupted teeth	3	1%
Surgical removal of a partially impacted tooth	1	0.3%
Surgical removal of an impacted tooth	6	1.7%
Surgical removal of a dental fragment	3	1%
Excision of the oral mucosa	1	0.3%
Incision and drainage of an oral abscess or cyst	34	10.8%
Intraoral suture	2	0.7%
Apical resection	1	0.3%
Excision of the maxillary or mandibular exostosis	1	0.3%
Unilateral mandibular ostectomy	19	6%
Unilateral maxillary ostectomy	17	5.3%
Maxillary osteotomy or osteoctomy with more than three procedures	2	0.7%

No., number; %, percentage.

**Table 5:** Cases evolution.

	Improved		Stationary		Healed	
	No.	%	No.	%	No.	%
2016	17	30.9%	0	0%	38	69.1%
2017	40	52%	1	1.3%	36	46.7%
2018	49	55%	0	0%	40	45%
2019	27	37.5%	2	2.8%	43	59.7%
2020	4	17.4%	0	0%	19	82.6%

No., number; %, percentage.

## Data Availability

The data presented in this study are available on request from the corresponding author.
